# Molecular basis for presentation of N-myristoylated peptides by the chicken YF1∗7.1 molecule

**DOI:** 10.1016/j.jbc.2025.110253

**Published:** 2025-05-22

**Authors:** Yogesh Khandokar, Tan-Yun Cheng, Carl J.H. Wang, Thinh-Phat Cao, Raghavendra S.K. Nagampalli, Komagal Kannan Sivaraman, Ildiko Van Rhijn, Jamie Rossjohn, D. Branch Moody, Jérôme Le Nours

**Affiliations:** 1 Infection and Immunity Program and Department of Biochemistry and Molecular Biology, Biomedicine Discovery Institute, Monash University, Clayton, Victoria, Australia; 2 Division of Rheumatology, Inflammation and Immunity, Brigham and Women’s Hospital, Harvard Medical School, Boston, Massachusetts, USA; 3 Faculty of Veterinary Medicine, Department of Infectious Diseases and Immunology, Utrecht University, Utrecht, The Netherlands; 4 Department of Medical Biology, Amsterdam University Medical Center, Amsterdam, The Netherlands; 5 Institute of Infection and Immunity, Cardiff University, School of Medicine, Heath Park, Cardiff, UK

**Keywords:** chicken, YF1∗7.1, immunity, MHC-Y, MHC, MDV, N-myristoylation, lipopeptides

## Abstract

Major histocompatibility complex I (MHC-I) and MHC-I-like molecules play a central role in mediating immunity. Through their conservation across all taxa of jawed vertebrates, the MHC-I-like proteins have adapted to present non-peptidic antigens to distinct T cell populations. While our understanding of the structure–function relationship of MHC-I and MHC-I-like molecules in humans and mice is well established, the nature of the antigens presented by MHC-I- like molecules in “non-model” species remains unclear. Here, using a mammalian recombinant expression system combined with mass spectrometry approaches, we identified N-myristoylated peptides as endogenous ligands for the chicken MHC-I-like protein YF1∗7.1. Given the importance of N-myristoylation in viral pathogenesis, we determined the crystal structure of YF1∗7.1 in complex with two N-myristoylated peptides derived from Marek’s disease virus (MDV), demonstrating the molecular basis that underpins the presentation of N-myristoylated peptides from MDV, a highly contagious and fatal viral neoplastic disease in chickens. Thus, the identified ligands are distinct from unmodified peptides found in classical MHC-I and -II as well as diverse amphipathic lipids captured by CD1 proteins. Collectively, our study lays the foundation for further molecular and functional characterization of YF1∗7.1 and more broadly of the role of the MHC-I encoded by the MHC-Y gene cluster in protection against highly contagious viral neoplastic diseases in chickens.

Over ∼400 million years of evolution, the immune system of jawed vertebrates adapted to survive in a variety of environments that are regularly exposed to bacterial, fungal, and viral threats. Central to the adaptive immune defense mechanism of jawed vertebrates are the major histocompatibility complex I and II (MHC-I and MHC-II) proteins. These protein bind pathogen-derived molecules known as antigens (Ags) to a highly diverse repertoire of T cell receptors (TCRs) that are expressed on the surface of T cells (1). Typically, MHC-I molecules present peptide-derived Ags to the TCRs for downstream T cell activation (2). In addition to classical MHC-I molecules, are MHC-I-like molecules, which are in some cases encoded outside the MHC gene locus and have clefts that are adapted to bind antigens that are distinct from linear peptides. For example, the MHC-I-related (MR1) molecule and the Cluster of Differentiation (CD1) family of proteins present small molecules and highly diverse lipid-derived Ags to specialized subsets of T cells, respectively (1, 3). In humans and mice, much progress has been made in understanding the molecular principles underpinning conventional MHC-I and MHC-I-like-mediated immunity (1). The recent availability of fully sequenced genomes of “non-model” species (4) has greatly contributed to a better understanding of how immunity operates in response to infections in species that include farm animals such as pigs, cows, and chickens. Yet, our understanding of the molecular correlates of this response is still extremely limited.

While the evolution of mammals and birds, including chickens, diverged more than 200 million years ago, the general features of both immune systems are remarkably conserved and include the genetic regions that encode for the MHC molecules (5). However, unlike the mammalian MHC region, the chicken MHC cluster of genes maps to two genetically unlinked regions localized on the same microchromosome (5), namely, the core MHC (MHC-B) and the secondary region (MHC-Y). The MHC*-*B locus encodes for only two classical MHC-I genes (BF1 and BF2), where BF2 is dominantly expressed (6, 7) and is associated with immune protection against viruses, including economically important viruses such as Rous sarcoma virus (RSV) (8). While the role of BF2 has been studied in the context of peptide-mediated immunity in chickens (9), much less is known about the MHC-Y region, also referred to as the Rfp-Y region. The MHC-Y region is located on chromosome 16 and contains a distinct set of genes that encode what appear to be classical MHC-I, MHC-II, and a variety of other proteins that may play roles in immune response and other functions (4).

Comparative studies suggested that the MHC-Y region has undergone significant evolutionary changes, possibly to adapt to specific immune challenges faced by chickens. Previous reports provided insights into an MHC-I encoded by one MHC-Y gene (YF1∗7.1), whereby the molecular analysis of an *in vitro* refolded YF1∗7.1 (10, 11) suggested that it may function as a lipid antigen-presenting molecule, similar to the CD1 family of proteins. However, the identity of the ligand(s) presented by YF1∗7.1 remained unknown.

Here, we produced a transmembrane truncated and secreted form of the YF1∗7.1 protein using a mammalian recombinant expression system, which allows protein capture in detergent free systems (3), as detergents can release ligands and mask mass spectrometry signals. Our sensitive mass spectrometry approach for detecting molecules eluted directly from YF1∗7.1 enabled the identification of N-myristoylated peptides as endogenous ligands for the chicken YF1∗7.1. N-myristoylation is a common lipid modification of proteins in many eukaryotes, consisting of the covalent attachment of a 14-carbon fatty acid (myristate) to the N-terminus of a protein *via* an amide bond formation (12).

These biochemical results define a new type of antigen in chickens that is distinct both from unmodified peptides bound to classical MHC molecules and from the diverse array of structurally unrelated amphipathic lipids presented by human and animal CD1 proteins. Further, these structures of YF1∗7.1-ligand binary complexes offer specific mechanistic insights into the mechanism of capture of N-terminally myristoylated peptides. Collectively, our study identified a ligand with a hybrid structure, including features of anchors for CD1 and peptides for classical MHC-I and II, setting the stage for the study of T cell response to myristoylated peptides, including common viral lipopeptides.

## Results

### Lipidomic analysis of ligands eluting from YF1∗7.1

**Figure 1:**
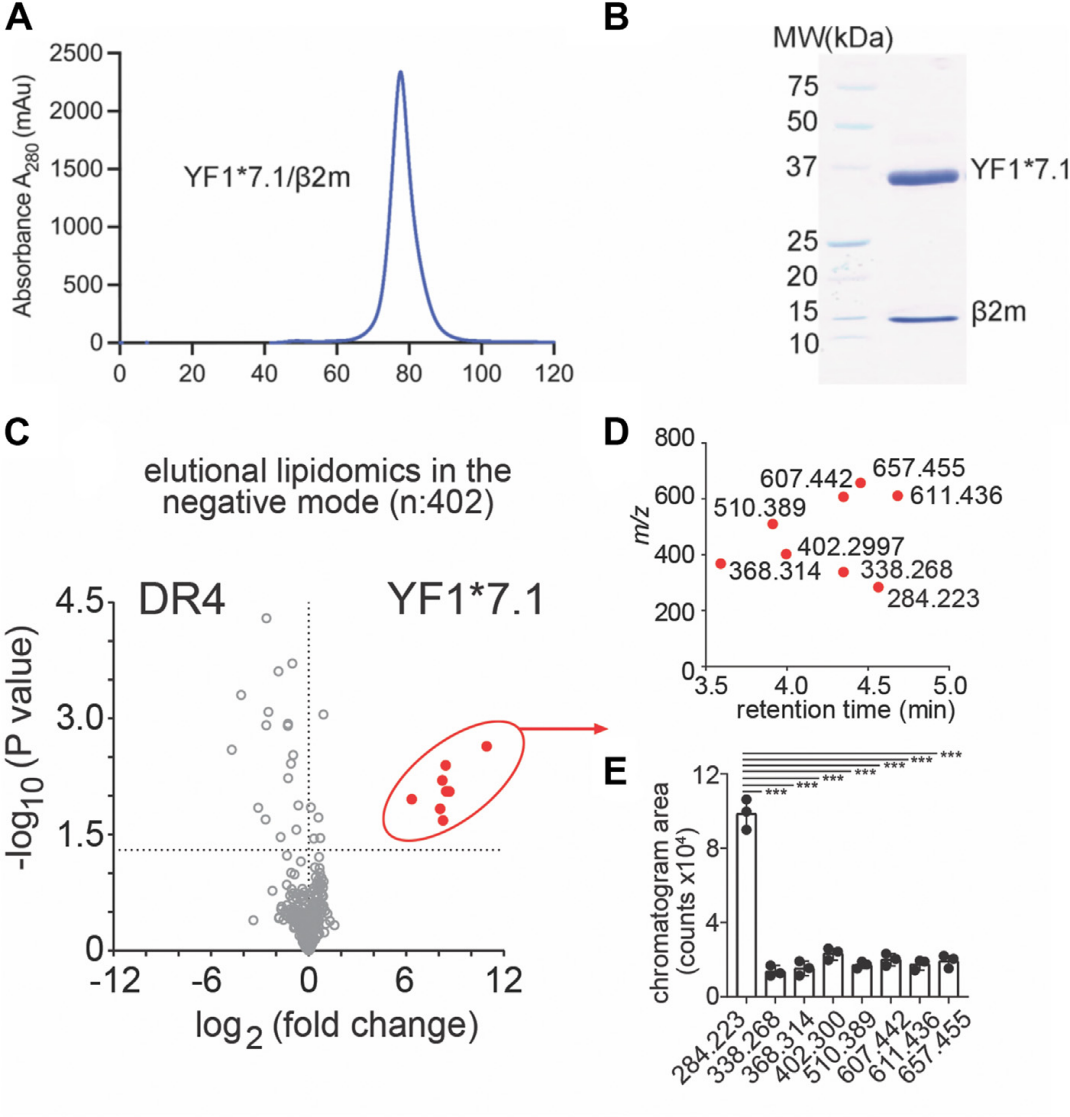
**Identification of YF1∗7.1 bound ligands by mass spectrometry.***A*, size exclusion chromatography (SEC) profile of the YF1∗7.1 protein using a mammalian expressions system (HEK293S). *B*, corresponding SDS-PAGE gel analysis of the SEC purified YF1∗7.1 protein. *C*, negative ion mode normal phase HPLC-QTOF-ESI-MS analysis of YF1∗7.1 and the HLA-DR4 (DR4) eluants detected 402 ions from which those with corrected *p* < 0.05 and fold-change > 50-fold are shown in *red*. *D*, plot of significantly changed ions shows clustering of molecules with similar masses (*m/z* 284.233) and retention time in a normal phase run. *E*, ion chromatogram areas of the eight unknown ions are shown in technical triplicate as the mean ± SD. The *p* values were calculated using the two-tailed Student’s-test. ∗∗∗ stands for *p* ≤ 0.0001.

Using a human embryonic kidney (HEK293S) cell line as a recombinant expression system, the chicken YF1∗7.1 protein was expressed in a secreted form and purified *via* immobilized nickel affinity chromatography (Ni-NTA) and size exclusion chromatography. The YF1∗7.1 molecule eluted at a volume corresponding to the molecular weight (∼43 kDa) expected for a correctly folded monomeric YF1∗7.1/β2m binary complex (Fig. 1, *A* and *B*). Next, the highly pure and soluble YF1∗7.1 protein complexes were precipitated in organic solvents, and lipids were extracted using a modified Bligh and Dyer extraction method that excludes proteins and captures cellular lipids (13). The eluted lipids were subject to normal phase high-performance liquid chromatography (HPLC) coupled with electrospray-ionization-Quadrupole-Time-of-Flight mass spectrometry (ESI-QTOF-MS) lipidomics system. Negative ion mode analysis of eluents extracted from YF1∗7.1 was compared with eluents from an equivalent amount of human MHC-II (HLA-DR4) used as a control protein (Fig. 1*C*). In this elutional lipidomics analysis, each unnamed molecule is called a feature, which is a datapoint with linked *m/z*, retention time, and intensity values. As contrasted with recent studies of human CD1 molecules that detected many hundreds or thousands of CD1-specific features extracted into organic solvents (3), only eight features showed YF1∗7.1 specificity *versus* the MHC-II, with a corrected *p* value < 0.05 and high fold-change > 50 (Fig. 1*C*, red). These data suggested that YF1∗7.1 might bind lipids but captured many fewer types of cellular lipids compared with human CD1 antigen-presenting molecules.

### Identification of YF1∗7.1-specific lipids as glycine lipids

**Figure 2:**
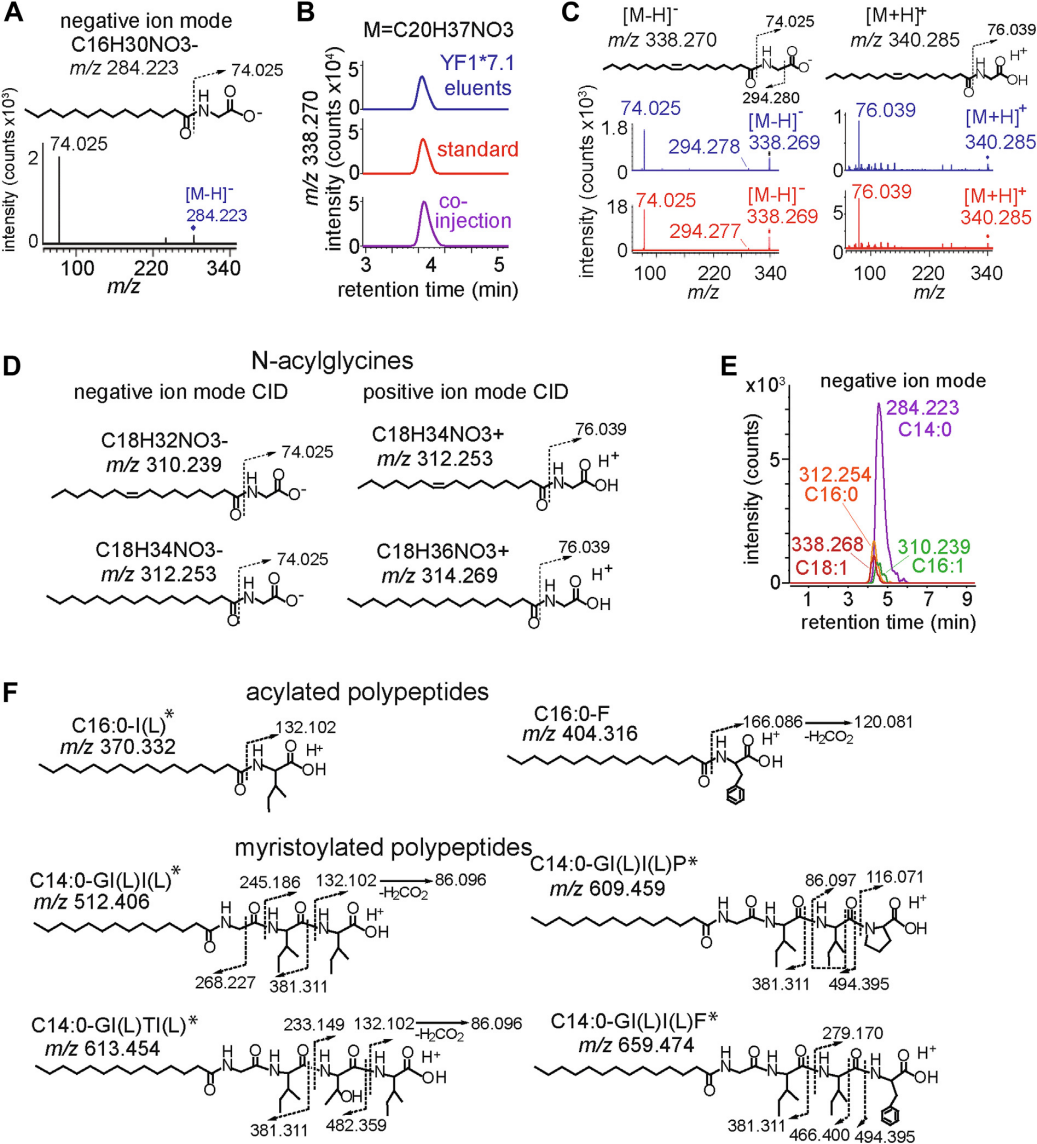
**Mass spectrometry fragmentation.***A*, identification of a glycine fragment (*m/z* 74.025) cleaved from *m/z* 284.223 or *m/z* 338.268 in the negative ion mode using collision-induced dissociation (CID)-MS. *B–C*, the identification of the natural YF1∗7.1 eluted compound with *m/z* 338.268 (*blue*) was determined by matching detected masses of the unknown, as well as co-elution and collision patterns with the synthetic standard, C18:1-glycine (*red*). *D*, N-acylglycines with the indicated acyl chains could be identified in the positive and negative mode. *E*, N-acylglycines with distinct alkyl chains that were eluted from YF1∗7.1 were detected in the negative mode with differing abundance but similar elution time on HPLC-MS. *F*, the positive ion mode CID-MS of eight unknown ligands solved the indicated lipopeptide structures. The amino acid sequence in each lipopeptide was determined by collision fragmentation. ∗indicates the presence of leucine or isoleucine, which cannot be distinguished by MS.

Further analysis of the features in a *m/z versus* retention time plot showed masses ranging from *m/z* 284.233 to *m/z* 657.455. However, all molecules had an early retention time (∼3.5–4.7 min) in a normal phase run, suggesting that the lipids were apolar (Fig. 1, *D* and *E*). This result also contrasted with the human CD1 lipidome, where the many eluted ligands showed markedly different retention times, where signals suggested markedly differing polarity of molecules, ranging from hydrophobes to polar phosphoglycolipids, suggesting again that the YF1∗7.1 capture pattern was more specific for ligands as compared to that of human CD1 proteins. Ion chromatogram areas provided initial insight into the relative abundance of each molecule, which revealed that the highest integration signal corresponded with the event at *m*/*z* 284.223, which was ∼5-fold more intense compared to the other seven remaining ions (Fig. 1*E*). Negative mode collision-induced dissociation (CID)-MS of this significant ion identified an ion consistent with glycine (*m/z* 74.025) and a mass interval consistent with myristate (C14:0) (Fig. 2*A*). Thus, the ion with strongest MS signal among YF1∗7.1 specific eluates was likely the well-known and conserved lipid anchor present in N-myristoylated peptides, myristoyl glycine (Fig. 2*A*). The cellular process of myristoylation favors myristoyl glycine conjugation, but other longer fatty acids are typically transferred at lower rates due to being poor substrates for N-myristoyltransferase (14). Indeed, a second lipidomics hit (*m/z* 338.268) corresponded to the mass of oleoyl-glycine, for which authentic standards were available (Fig. 2*B*). Further analysis of the molecule released from YF1∗7.1, and the authentic standard showed precise chromatographic co-elution as well as release of the proposed glycine ion, *m/z* 74.025 in the negative and *m/z* 76.039 in the positive mode (Fig. 2*C*), unambiguously establishing its structure. The other six primary hits that met objective statistical change criteria did not match the mass of additional N-acylglycine family members (Fig. 1*C*). However, targeted analysis of the lipidomic dataset, which included ions with low intensity or those that did not meet predetermined thresholds for fold-change, detected further ions in the lipidome that match the expected patterns of other naturally occurring acyl glycines, including palmitoleoyl (*m/z* 310.239) and palmitoyl (*m/z* 312.254) glycine, where both proposed structures were further confirmed through CID-MS in both the positive and the negative mode (Fig. 2*D*). Considering both targeted and untargeted MS, these results identified the brightest YF1∗7.1 ion as palmitoylation substrate C14:0 glycine, and additional family members with C16, C16:1, and C18:1 with lower signals, matching known biological patterns of “myristoylation” substrates (Fig. 2*E*). These findings highlight YF1∗7.1's specificity for N-myristoylated glycine and related acylglycine, with C14:0 glycine as the predominant ligand, aligning with established patterns of myristoylation substrates.

### Identification of structurally related myristoylated peptides

We hypothesized that the other 7 YF1∗7.1-specific unknowns from the untargeted lipidomics “hit list” (Fig. 1*C*) might be myristoylated polypeptides. This perspective allowed identification of the remaining six ligands as protonated adducts in positive mode CID-MS (Fig. 2*F*). Four of the structures were N-myristoylated peptides with three or 4 amino acid residues. The [M+H]^+^ ion *m*/*z* 512.046 matched the sequence N-myristoyl-glycine-isoleucine (or leucine)-isoleucine (or leucine) (C14:0-GI(L)I(L)), whereas *m*/*z* 609.459 matched C14:0-GI(L)I(L)P, *m*/*z* 613.454 was C14:0-GI(L)TI(L), and m/z 659.474 was C14:0-GI(L)I(L)F. We further identified two N-palmitoylated peptides, C16:0-I(L) at *m/z* 370.332 and C16:0-F at *m/z* 404.316 (Fig. 2*F*). These identifications accounted for all eight of the high-fold change hits seen in the negative mode lipidomics (Fig. 1*C*). MS analysis did not allow differentiation of leucine from isoleucine, but otherwise the structures were assigned based on diagnostic CID fragments (Fig. 2*F*). Contrasting with the known patterns of unmodified peptide eluting from classical MHC proteins and diverse lipids eluting from CD1, our lipidomic analysis of the mammalian expressed YF1∗7.1 identified a novel chemical class of structurally related bound ligands suggesting that YF1∗7.1 captures N-acylglycine groups involved in myristoylation reactions, as well as extended “myristoylated” peptides. The acyl chains ranged from 14 to 18 carbons in length and the peptides varied in length from 1-mer to 4-mer. These findings highlight the unique ligand-binding capabilities of YF1∗7.1, expanding our understanding of its role in capturing structurally diverse N-acylglycine and myristoylated peptides.

### Molecular presentation of N-myristoyl-glycine by the chicken YF1∗7.1

**Figure 3:**
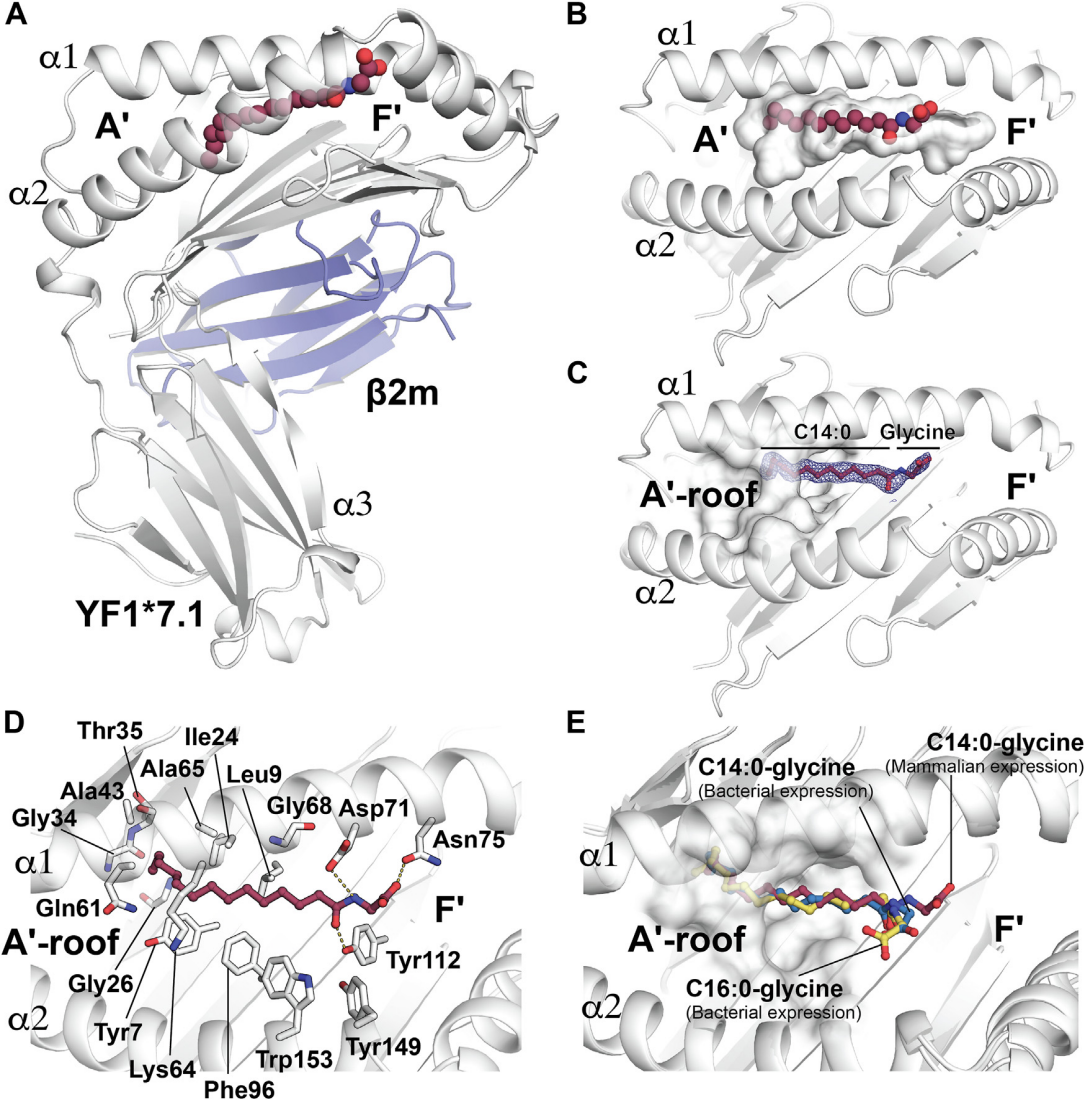
**Molecular insights into the presentation of endogenous N-myristoylated lipopeptides by YF1∗7.1.***A*, crystal structure of the mammalian cell produced chicken YF1∗7.1. YF1∗7.1, *light grey*; β2-microglobulin (β2m), *blue*. The bound N-myristoyl-glycine is depicted as *spheres*. *B*, *top* view of the YF1∗7.1 binding groove (*white surface*) with the bound N-myristoyl-glycine (*spheres*). *C*, top view of the YF1∗7.1 binding groove with the 2Fo-Fc electron density map (0.75 σ level) of the bound N-myristoyl-glycine (*red ball and sticks*). *D*, molecular interactions of N-myristoyl-glycine with YF1∗7.1. Hydrogen bonds are shown in *yellow dashed lines*. *E*, superposition of YF1∗7.1 with the bound N-myristoyl-glycine (C14:0) (in *red*) from mammalian cell expressed YF1∗7.1, N-myristoyl-glycine (C14:0) (in *blue*) from bacterially expressed YF1∗7.1 (*in vitro* refolded) and N-myristoyl-glycine (C16:0) (in *yellow*) from bacterially expressed YF1∗7.1 (*in vitro* refolded).

Next, to gain insight into the molecular presentation of the N-myristoylated lipopeptides by YF1∗7.1, we determined the crystal structure of YF1∗7.1 to 2.0 Å resolution that was produced in the same mammalian expression system as for the mass spectrometry analysis (Fig. 3*A* & Table S1). The native YF1∗7.1 protein adopted a very similar overall structure compared to that of YF1∗7.1 that was produced in *Escherichia coli* and refolded *in vitro* (11) with a root mean square deviation (rmsd) of ∼0.6 Å over 313 Cα (Fig. S1*A*). YF1∗7.1 exhibited the typical architecture of MHC-I and MHC-I-like molecules in which the heavy chain was composed of the α1-and α2-helices that formed the antigen binding groove and an α3-domain that was non-covalently associated with β2-microglobulin (β2m) (Fig. 3*A*). As previously described in (11), the YF1∗7.1 antigen-binding cleft is composed of two distinct pockets that differed in size and shape (Fig. 3*B*) and are termed A′- and F′-pockets analogous to the established nomenclature originally established for CD1 pockets (15). The high resolution of the YF1∗7.1 crystal structure enabled identification of a clear electron density emerging from the binding groove of YF1∗7.1 (Fig. S1*B*). Our ESI-MS analysis of the produced YF1∗7.1 suggested that N-myristoyl-glycine (C14:0-gly) was the most abundant ligand within the YF1∗7.1 soluble preparation, so this ligand was then modeled within this residual electron density. The structure of the YF1∗7.1-C14:0-gly binary complex was further refined (Table S1) and confirmed that the C14:0-gly as the bound ligand within the YF1∗7.1-binding groove (Fig. 3*C*). This result revealed that the eluted lipopeptides were likely derived from the YF1∗7.1 binding cleft, and it provided specific structural insights into how lipopeptides with differing alkyl chain length and peptide length could be captured.

Here, the C14:0 acyl chain was sequestered within the A′-pocket, whilst the terminal glycine headgroup was positioned towards the F′-pocket of the binding groove (Fig. 3*C*). The overall positioning of the C14:0-gly myristoylated lipopeptide was similar to that of the polyethylene glycol (PEG) molecule (Fig. S1*A*) identified in the *in vitro* refolded YF1∗7.1 crystal structure (11). The C14:0 acyl chain occupied ∼70% of the A′-pocket volume and contacted the hydrophobic residues Tyr7, Leu9, Ile24, Ala43, Phe96, Tyr149 and Trp153 of YF1∗7.1 *via* van der Waals interactions (Fig. 3*D* & Table S2). The carbonyl oxygen of the myristate moiety was hydrogen bonded to Tyr112. The main chain N and O of the Gly residue formed hydrogen bonds with Asp71 and Asn75, respectively (Fig. 3*D* & Table S2). ∼90% of the C14:0-Gly was buried within the YF1∗7.1 binding groove with a buried surface area of ∼530 Å^2^, leaving ∼10% of its molecular surface potentially (Fig. 3*B*) exposed for direct recognition by a cognate receptor, similar to the presentation of headless lipid-based antigen by the CD1a molecule in humans (16) that is a close structural homolog of YF1∗7.1 with rmsd of ∼1.9 Å over 134 Cα.

To further ascertain the ability of the chicken YF1∗7.1 to bind N-myristoyl-Gly ligands, the protein was recombinantly expressed in *E. coli* and refolded *in vitro* in the presence of the most abundant N-myristoyl-glycine (C14:0) (*m/z* 284.223) identified from the mass spectrometry analysis of the YF1∗7.1 elution (Fig. 1, *C*–*E*) and of a N-palmitoyl-glycine that contains a longer acyl chain (C16:0). Next, we determined the crystal structure of both binary complexes (Table S1) that also revealed unambiguous electron density for both C14:0-gly and C16:0-gly lipopeptides (Fig. S1, *C* and *D*). The C14:0 and C16:0 acyl chains of both ligands were located within the A′-pocket in a very similar location to that of the endogenously bound mammalian N-myristoyl-glycine (C14:0) sharing similar contacts with YF1∗7.1 (Table S3). The glycine exhibited slightly different orientations within the binding groove (Fig. 3*E*). These structural analyses provide crucial insights into the binding mechanism of N-myristoyl glycine into the YF1∗7.1 binding groove.

### Virus-derived myristoylated epitopes presented by the chicken YF1∗7.1

**Figure 4:**
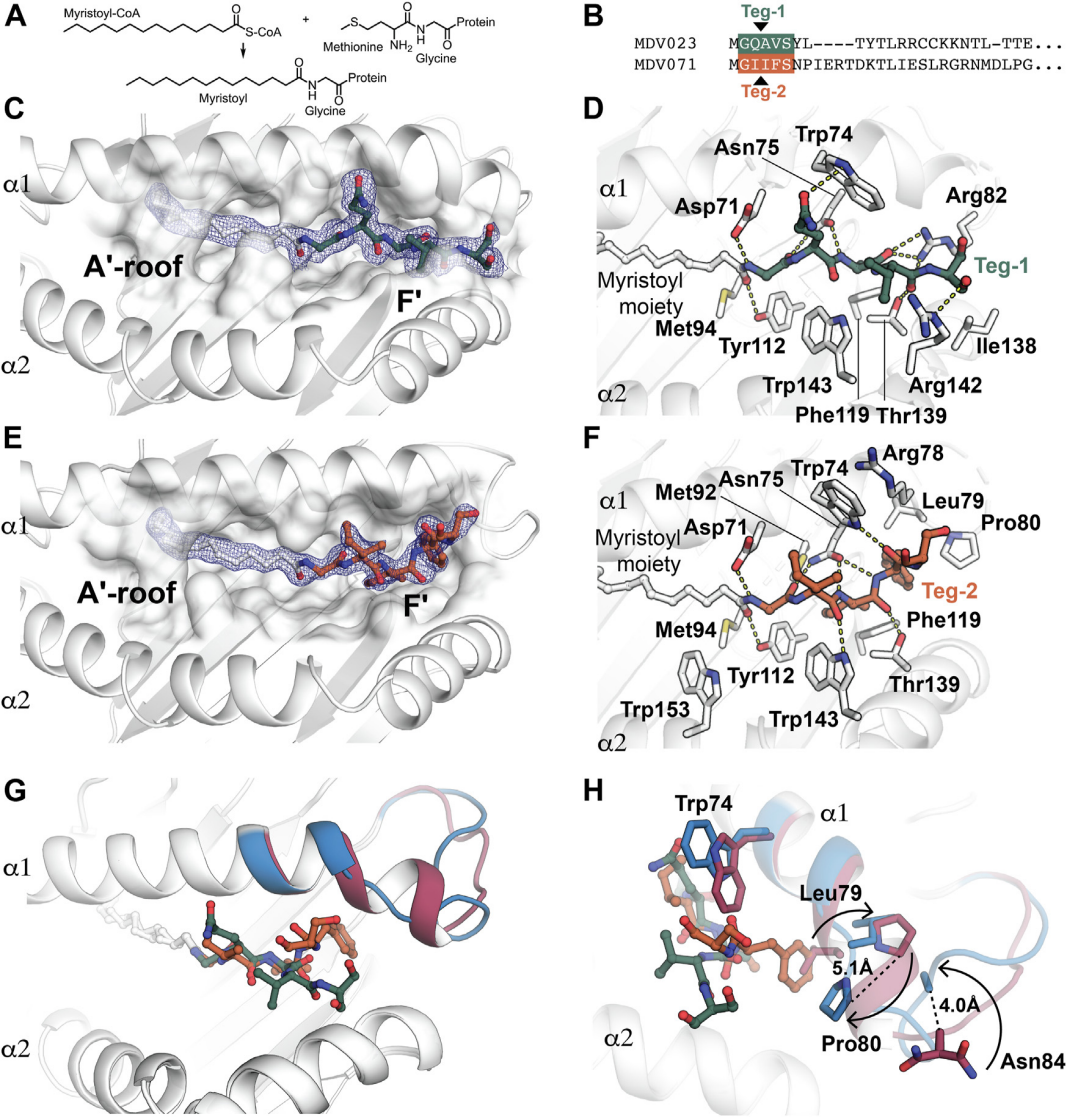
**Molecular presentation of MDV derived N-myristoylated lipopeptides by YF1∗7.1.***A*, schematic representation of the N-myristoylation reaction is shown. *B*, amino acid sequences of MDV tegument proteins (Teg-1 and Teg-2) that can be fused to the C14:0 myristoyl moiety are shown. *C*, *top* view of the YF1∗7.1 binding groove with the 2Fo-Fc electron density map (0.75 σ contour level) of the bound C14-teg-1 in YF1∗7.1. *D*, molecular interactions of C14-teg-1 with YF1∗7.1 are shown. Hydrogen bonds are shown as *yellow dashed lines*. *E,top* view of the YF1∗7.1 binding groove with the 2Fo-Fc electron density map (0.75 σ contour level) of the bound C14-teg-2 in YF1∗7.1. *F*, molecular interactions of C14-teg-1 with YF1∗7.1. Hydrogen bonds are shown in *yellow* as dashed lines. *G*, superposition of the YF1∗7.1-C14-teg-1 and YF1∗7.1-C14-teg-2 structures depicted as cartoons. The C-terminal end of the α1-helix of the YF1∗7.1-C14-teg-1 and YF1∗7.1-C14-teg-2 structures are coloured in *blue* and *magenta*, respectively. *H*, structural rearrangement of residues at the C-terminal end of the α1-helix between YF1∗7.1-C14-teg-1 (residues in *blue*) and YF1∗7.1-C14-teg-2 (residues in *magenta*).

Viral proteins are commonly myristoylated and this post-translational modification is strongly linked to pathogenesis (17) whereby the N-myristoyltransferase (NMT) catalyses the condensation of 14-carbon fatty acid myristate moiety to the N-terminal glycine of proteins (Fig. 4*A*). For instance, in chickens, tegument proteins from the highly contagious and fatal viral neoplastic Marek’s disease virus (MDV) were susceptible to N-myristoylation upon viral infection (17, 18, 19). We hypothesized that YF1∗7.1 may also play a role in the presentation of MDV-derived N-myristoylated peptide epitopes Here, our search and analysis of sequences for the N-myristoylation motif (MGXXXS/T) within the MDV genome resulted in the identification of two tegument proteins (Uniprot ID: MDV023 (teg-1) and MDV071 (teg-2)) as potential candidates for myristoylation (18). As proof of concept, we then selected and synthesized 5-mer amino acid long peptides from teg-1 (GQAVS) and teg-2 (GIIFS) post myristoylation motif (GXXXS/T) (Fig. 4*B*) to investigate their molecular presentation by the chicken YF1∗7.1. Next, both teg-1 and teg-2 peptides harbouring a C14:0 acyl chain were commercially synthesized, and YF1∗7.1 was refolded in *vitro* in the presence of the individually synthesized MDV-derived M-myristoylated peptide epitopes. The resultant YF1∗7.1-teg-1(C14:0) and YF1∗7.1-teg-2 (C14:0) binary complexes were purified using chromatographic procedures, crystallized, and their structure was determined to 2 Å and 1.8 Å resolution, respectively (Table S1). The residual electron density accounting for the N-myristoylated teg-1 (C14:0) and teg-2 (C14:0) peptides was unambiguous (Fig. S2), enabling accurate modeling of both ligands (Fig. 4, *C* and *D*). In both binary complexes, the C14:0 acyl chain and the glycine residue adopted an overall position similar to that of the N-myristoyl-glycine (C14:0) within the A′-pocket, and the molecular contacts with YF1.7∗1 were largely conserved (Fig. 2 & Table S4) that included the three hydrogen bonds formed with Asp71, Asn75 and Tyr112. The similar positioning of the acylglycine anchor between the five binary complexes strongly suggests that the acylglycine, which is established as the core motif for protein myristoylation modifications, is also an anchor for the binding of differing lipopeptides in the pocket of YF1∗7.1.

Examining this interaction in more detail, within the YF1∗7.1-C14:0-teg-1 complex, the main chain of *P2-Gln* contacted Asp71, Trp74, and Asn75 *via* VDW interactions (Fig. 4D), whilst the side chain was solvent exposed (Fig. S3*A*) and interacted solely with Trp143. The *P3-Ala* was anchored deep within the F′-pocket by establishing main chain hydrogen bonds with Asn75 and Arg82 and side chain VDW contacts with Trp74, Asn75, Arg82, Phe119 and Thr139. The *P4-Val* backbone was buried within the pocket whilst the sidechain was entirely exposed to the solvent (Figs. 4*D* & S3*A*). *P4-Val* formed a main chain hydrogen bond with Thr139 while establishing VDW interactions with Arg82, Thr139, Arg142, and Trp143 (Fig. 4*D*). Finally, the *P5-Ser* side chain was also solvent exposed and interacted with Arg82, Ile138, and Arg142 (Fig. 4*D*).

In the YF1∗7.1-C14:0-teg-2 structure, the *P2-Ile* was partially exposed to the surface (Fig. S3*B*) by forming a hydrogen bond with Trp143 and establishing VDW interactions with Asp71, Trp74, Asn75, and Trp143 (Fig. 4*F*). The *P3-Ile* was deeply buried in the F′-pocket, where the main chain atoms formed hydrogen bonds with Asn75 and Thr139 whereas the side chain was involved in VDW interactions with Asn75, Met92, Phe119, Thr139, and Trp143 (Fig. 4*F*). Similarly, the bulky hydrophobic *P4-Phe* was fully embedded within the F′-pocket and its main chain formed two hydrogen bonds with Trp74 and Asn75, whereas the side chain established VDW interactions with Trp74, Asn75, Arg78, Leu79, Pro80, and Phe119. Similar to the YF1∗7.1-C14:0-teg-1 complex, the C-terminal residue *P5-Ser* was exposed to the surface (Fig. S3*B*) and engaged with Trp74 and Arg78 *via* VDW interactions. These results demonstrate that YF1∗7.1 effectively binds and presents N-myristoylated viral epitopes, highlighting its role in recognizing diverse lipopeptides and expanding our understanding of its molecular interactions with viral-derived ligands.

### Malleability of the YF1∗7.1-binding groove to accommodate 5-mer myristoylated peptides

In the crystal structure of the five YF1∗7.1-ligand binary complexes determined in the study, the overall architecture of the A′-pocket remained unchanged (Fig. 4*G*). The positioning of the residues Asp71, Asn75, and Tyr112 that contacted the myristate carbonyl and the *P1-Gly* residue was also conserved by anchoring the myristoyl moiety and the Gly residue within the binding groove. However, to accommodate the two distinct teg-1 and teg-2 lipopeptides, the F′-pocket of YF1∗7.1 exhibited structural malleability (Fig. 4*H*). Here, relative to the YF1∗7.1-C14:0-teg-1 complex, the last turn of the α1-helix and following loop (Leu79 to Asn84) in the YF1∗7.1-C14:0-teg-2 complex underwent significant structural rearrangement (Fig. 4*H*) to accommodate the bulkier *P4-Phe* whereby Trp74 and Leu79 adopted distinct conformations to avoid steric clashing with the *P4-Phe* and *P5-Ser*, respectively. No other significant movements were observed within the α-helices of the two binary complexes. The overall pattern observed for bound acylglycines and glycine lipopeptides was that the acyl chain, which is similar in all molecules, and was inserted into the A′-pocket, where it could promote anchoring and is protected from exposure to the aqueous exterior solvent by the A′-roof, a structural modification seen in human CD1 but not MHC I proteins. In contrast, the peptide region of the lipopeptide, namely, the *P2-P5* region, were exposed outside the F′-pocket for possible recognition, or were partially inserted into the F′-pocket.

### Shared YF1∗7.1 molecular features across the chicken MHC-Y genes cluster

**Figure 5:**
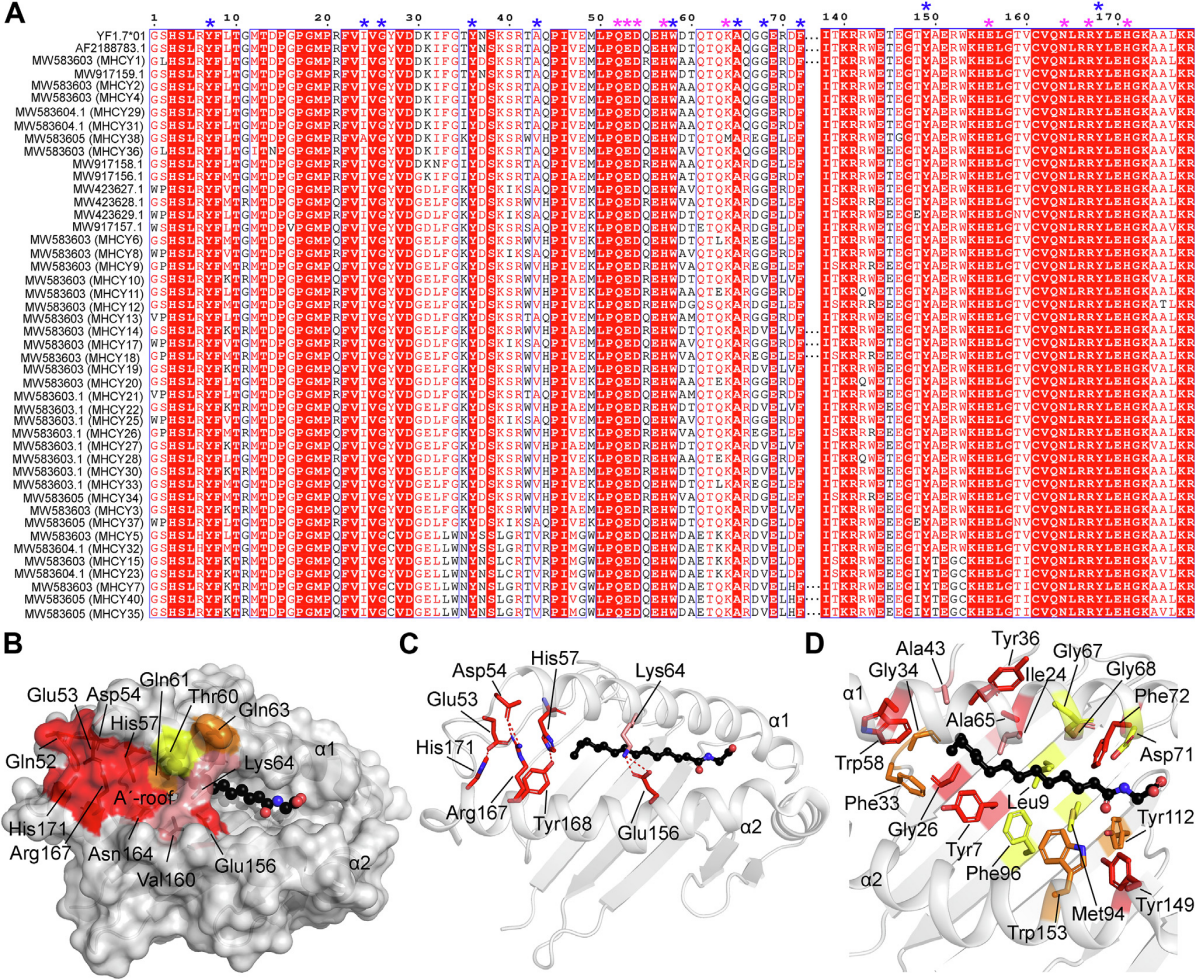
**YF1∗7.1 molecular features across the MHC-I proteins encoded by the MHC-Y cluster of genes in chicken.***A*, sequence alignment of the chicken YF1∗7.1 and MHC-Y proteins. For clarity, only part of the alignment is shown (Residues 1–72 and 138–178). The strictly conserved residues are shown on a *red* background. Similar residues are shown in *red* characters and similarity across the sequences are indicated with a *blue* frame. The program Clustal Omega (57) was used to generate the alignment that was edited using ESPript 3.0 (58). A′-roof residues that are >90% identical or 100% similar are indicated with a *magenta* star above the alignment. A′-pocket residues contacting the N-myristoyl glycine in YF∗7.1 that are >90% identical or 100% similar are indicated with a *blue* star above the alignment. *B*, surface representation of YF1∗7.1 with the bound N-Myristoyl glycine (C14:0) shown as *black spheres*. A′-roof residues that are fully conserved (in *red*), 90% identical or 100% similar (in *salmon*), >80% similar (in *orange*) and <80% similar (in *yellow*) across all MHC-Y class I sequences. *C*, A′-roof molecular contacts. Hydrogen bonds are shown as *yellowdashes*. *D*, *cartoon* representation of the A′-pocket of YF1∗7.1 with the bound N-Myristoyl glycine shown as *black spheres*. A′-pocket residues contacting N-myristoyl glycine (C14:0) *via* their sidechain that are fully conserved (in *red*), 90% identical or 100% similar (in *salmon*), >80% similar (in *orange*) and <80% similar (in *yellow*) across all MHC-Y class I sequences.

Over the years, the high number of repetitive sequences and the presence of multiple gene family members have hindered the accurate assembly of short-read sequence data for the MHC-Y cluster of genes such that these genes have therefore been underrepresented in genomic sequence data. However, using single-molecule real-time sequencing of BAC clones for the *Gallus gallus* Red Jungle Fowl reference genome, Goto *et al.* (20) identified 107 MHC-Y region genes that encoded 45 MHC molecules. Thus, the availability of these gene sequences offered an opportunity to gain insights into the possible conservation of the unique molecular features exhibited by YF1∗7.1 across the MHC-Y region in ways that may contribute to understanding the chemical classes of ligands presented by these encoded proteins. Here, our sequence analysis of the α1-α2 domain of the YF1∗7.1 against the 45 MHC-Y sequences revealed a very high degree of conservation of the 13 residues forming the A′-roof to the YF1∗7.1 molecule (Fig. 5*A*). Over 75% of the A′-roof residues (Gln52, Glu53, Asp54, His57, Lys64, Glu156, Val160, Asn164, Arg167 and His171) were >90% conserved or 100% similar within the 45 sequences analyzed (Fig. 5, *A* and *B*). Here, only one sequence exhibited a Lys64Met mutation whilst Val160 was replaced by a similar residue (Ile) in five of the sequences. Interestingly, Lys64 formed two hydrogen bonds with Glu156 that may play a key role in the A′-roof formation by tethering both α1-and α2-helices to one another (Fig. 5*C*). Gln61 and Gln63 were either conserved or replaced by a similar residue (Glu) in >80% of the sequences (Fig. 5, *A* and *B*). In the A′-pocket, ∼55% of the residues contacting the N-myristoyl moiety (C14:0) in YF1∗7.1 (Tyr7, Ile24, Gly26, Tyr36, Ala43, Trp58, Ala65, Gly 68, Phe72, and Tyr149) were >90% conserved or 100% similar (Fig. 5, *A* and *D*). These data suggest that the A′-roof structures, which are shown here to cover and block external presentation of the acyl chains to the outer surface, may be evolutionarily conserved among proteins encoded by MHC-Y loci, as also seen for human CD1 proteins (21).

### Molecular presentation of N-myristoylated lipopeptides across species

**Figure 6:**
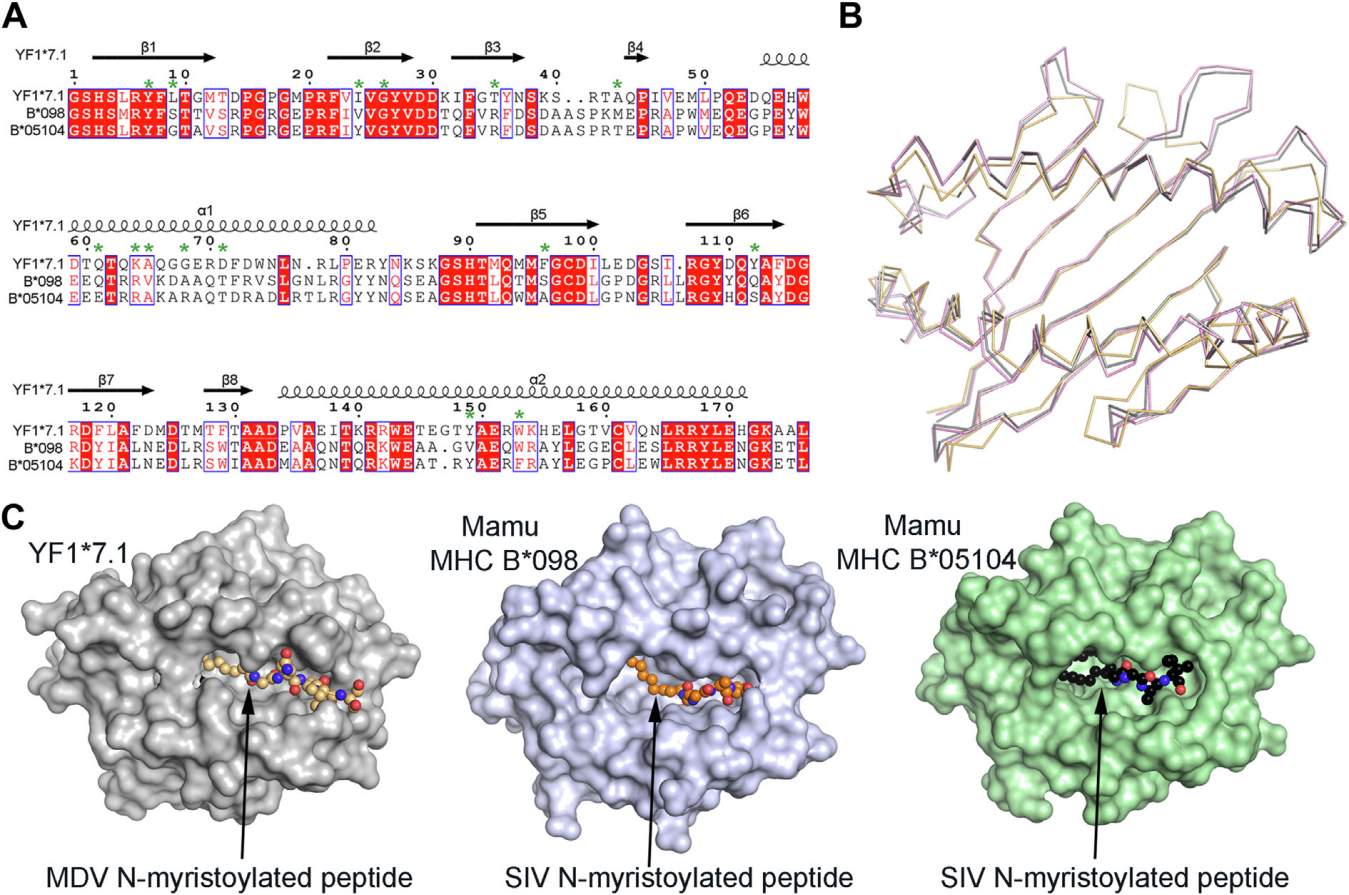
**Molecular comparison of the chicken YF1∗7.1 and primate MHC proteins presenting N-myristoylated lipopeptides.***A*, structure-based alignment of the chicken YF1∗7.1 and the primate Mamu B∗098 (Pdb code: 4ZFZ) (23) and B∗05,104 (Pdb code: 6IWG) (25). The strictly conserved residues are shown on a *red* background. Similar residues are shown in *red* character and similarity across the three sequences are indicated with a *blue* frame. Secondary structural elements of the YF1∗7.1 crystal structure are shown atop the alignment and the numbering is based on the YF1∗7.1 amino acid sequence. Residues involved in contacting the myristate moiety of N-myristoylated glycine are marked as *green* stars. The program Indonesia was used to generate the structure-based sequence alignment that was edited using ESPript 3.0 (58). *B*, superposition of the α1/α2 domain of YF1∗7.1 (*orange*), Mamu MHC B∗098 (Pdb code: 4ZFZ) (*grey*) (23) and Mamu MHC B∗05104 (Pdb code: 6IWG) (*pink*) (25) shown as *ribbons*. *C*, surface representation of YF1∗7.1 (*gray*), Mamu MHC B∗05104 (*light blue*) and Mamu MHC B∗098 (*green*) with bound N-myristoylated lipopeptides shown as *spheres*.

Similarly, in non-human primates’ viruses such as in the Simian Immunodeficiency Virus (SIV), the negative regulatory factor (Nef) protein can be N-myristoylated. Previous studies reported that MHC-I (Mamu-B∗098 and Mamu-B∗05,104) from the *Rhesus macaques* also evolved to present N-myristoylated lipopeptides (4- to 5-*mer*) that were derived from the simian immunodeficiency virus (SIV) Nef protein to subset of T cells (22, 23). While the chicken YF1∗7.1 shared a low sequence identity (∼40%) with the Mamu-B∗098 and B∗05,104 counterparts (Fig. 6*A*), the α1/α2-superdomain architecture of YF1∗7.1 was very similar to that of the Mamu MHC-I with a rmsd of 1.3 Å over ∼160 Cα atoms (Fig. 6*B*). YF1∗7.1 and Mamu MHC-I exhibited a more open binding cleft architecture (Fig. 6*C*) relative to YF1∗7.1. Within the A′-pocket, ∼30% of the YF1∗7.1 residues (Tyr7, Gly26, Gln61, Lys64, and Ala65) that contacted the myristate moiety of the lipopeptide were conserved or similar within the Mamu MHC-I. Due to the presence of bulkier hydrophobic residues (Trp153, Leu9, Phe96, Tyr112, and Trp153), the A′-pocket of YF1∗7.1 was more restricted than that of the Mamu MHC-I. Interestingly, Lys64 was replaced by an Arg residue (Arg66) in the Mamu MHC-I B∗098 and B∗05,104. This residue formed hydrogen bonds with Glu163 within the α2-helix of B∗098, tethering both helices, enabling the formation of a similar A′-roof to that of YF1∗7.1.

## Discussion

MHC-I and MHC-I-like molecules are pivotal to the vertebrate immune system's ability to recognize and respond to pathogens. The evolution and conservation of these molecules across different species highlighted their essential role in immunity. MHC-I molecules are well-known for presenting peptide-derived antigens to cytotoxic T cells that then eliminate infected cells. Yet, whilst similar in their overall architecture, MHC-I-like molecules, encoded outside the MHC gene locus, or non-classical MHC proteins within the MHC cluster, can present a broader range of antigens beyond unmodified linear peptides that include lipids and small molecules to specific subsets of immune cells (24). This versatility can generate TCR-mediated recognition events that can discriminate self from foreign or otherwise allow response to infection. Our study primarily focused on exploring the repertoire of ligands that may be presented by the understudied chicken MHC-Y cluster of genes that encodes for MHC-I molecules based on their amino acid sequence analysis. While the outcome of YF1∗7.1-mediated immune responses awaits development of new systems to interrogate avian immune systems, the discovery of YF1∗7.1 ligands and demonstration of their binding patterns provides substantial insight into candidate structural mechanisms of antigen recognition in patterns that are intermediate between well-studied peptides and lipid-based antigen presenting molecules.

Detergents needed for capturing of transmembrane molecules disrupt bound lipids from the antigen binding cleft and strongly mask MS signals. Therefore we, like many prior studies in the CD1 field, produced transmembrane truncated proteins to overcome these artifacts. One recent study of human CD1 proteins showed strong overlap of lipid repertoires arising from transmembrane truncated proteins *versus* enzyme-cleaved transmembrane constructs (3). This new approach suggests that transmembrane truncation does not cause major artifacts and would be a conceivable experiment for follow-up studies on YF1∗7.1.

Here, we demonstrated that, as for the Mamu MHC-I (22, 23, 25) in primates, the binding groove of the chicken YF1∗7.1 protein has evolved to present N-myristoylated peptides by sharing similar structural features of the chicken CD1 (26, 27) and MHC-I (28, 29, 30, 31, 32) and thus expanding the repertoire of ligands beyond peptides and lipids that can be presented by the classical MHC-I and the CD1 (chCD1-2) molecules in chickens, respectively.

Further, while no method can formally rule out future discovery of additional lipids bound to YF1∗7.1, the spectrum of ligands detected by unsupervised lipidomics points to differences between YF1∗7.1’s and CD1’s Ag capture with regard to the number of ligands and their chemical diversity, where the former is specific for myristoylation products and CD1 captures most major membrane lipids. Typically, myristoylation occurs post-translationally and plays critical roles in viral pathogenicity and replication, where it often serves to target and anchor proteins to cellular membranes in viral assembly and budding processes. Viruses lack N-myristoyltransferase, but foreign proteins are captured by mammalian enzymes for efficient generation of foreign lipopeptides in cells (17). By modifying the structural or regulatory proteins of the virus, myristoylation can also influence the various stages of the viral life cycle, such as entry, replication, assembly, and egress (17). Considering the importance of N-myristoylation in viral processes and the susceptibility of chickens to a variety of highly pathogenic viruses, including MDV, RSV, and influenza, we therefore hypothesize that this modification might be a signal for immune recognition in chickens *via* the presentation of viral lipopeptides by the MHC-I-like molecule YF1∗7.1.

Recent molecular insights into the comparison of the classical MHC-I proteins with CD1 molecules have suggested that the A′-roof was the key evolved structural feature that distinguished these two families of antigen-presenting molecules (21) from classical MHC-I and MHC-II, which lack roofs. The A′-roof closes off the membrane distal surface of the human CD1 proteins to create a deeper cleft and solvent non-exposed environment, as well as a large unliganded surface for TCR binding. This structural analysis, with the universal presence of A′-roofs in CD1 gene-encoded lipid antigen-presenting molecules *versus* the absence of A′-roofs in MHC-I encoded peptide-presenting molecules, suggested that the A′-roof was the key evolved structural feature that allows lipid capture (21).

Here, the presence of an A′-roof in the chicken YF1∗7.1 molecule, along with the high degree of sequence conservation within the A′-roof and A′-pocket among the majority of the 45 encoded MHC-Y molecules observed, suggested that this cluster of MHC-I genes may have evolved to present a similar class of antigens, namely, lipopeptides rather than the typical peptides. In turn, this finding suggested the hypothesis that lipopeptide-antigen representative molecules emerged at least three times during bird-mammal evolution through the emergence of MHC-Y, Mamu-MHC, and CD1 molecules. The A′-roof conservation analogous to the CD1 system allowing lipid-independent recognition (33, 34) could suggest that there may also be ligand-independent recognition of YF1∗7.1 by autoreactive TCRs in contrast to the co-recognition paradigm for TCR-MHC-peptide. It remains to be determined whether the recognition mechanism of YF1∗7.1 will be peptide sequence specific, as in the MHC-I and -II systems, or A′-roof centric. Further, given the ‘non-classical’ nature of YF1∗7.1, there may also be invariant TCR usage directed against YF1∗7.1, similar to NKT and MAIT cells' recognition of CD1d and MR1, respectively (21). However, CD1d and MR1 are also ligands for more diverse T cell subsets (35, 36, 37, 38, 39, 40), so it remains to be determined whether invariant or diverse TCR usage predominates for the YF1∗7.1 molecule.

Overall, the ligand specificity of these three non-polymorphic lipid binding proteins is not likely equivalent. Data reported suggest that the lipid binding cleft volume of YF1∗7.1 is approximately one-third of the human CD1a or CD1c (3). Corresponding to this smaller lipid size, we detected only singly acylated myristoyl ligands in this lipidomics approach, whereas most cellular ligands captured by the four human CD1 proteins are diacylglycerols or sphingolipids with two alkyl chains (3, 41). In agreement with preference for dual *versus* single chain lipids, mass spectrometry analysis of *in vitro* refolding experiments in the presence of lipid extract from *E. coli*, an earlier report suggested single chain lysophospholipids such as the lyso-phosphatidylethanolamine (lyso-PE) (18:1) as a dominant bound ligand for two MHC-Y class I isoforms (YF1∗*7.1 and YF1*RJF34) (42). While the spectrum of human CD1 ligands is dominated by dual-chain lipids, at least some CD1 ligands can have single alkyl chains, so that the "lipid anchor" is smaller than the cleft. The CD1a molecule exhibited dual ability to present a spectrum of lipid-based antigens and a single lipopeptide known as didehydroxymycobactin that was derived from *Mycobacterium tuberculosis* (PDB ID: 1XZ0) (43). Also, CD1c can present both phospholipids and a glycine acylated lipopeptide that is related in structure to lipopeptides reported here (44).

Observed similarities in sequences from the loaded MDV lipopeptides (myr-GIIFS) and several of the endogenously captured lipopeptides reported here (myr-GII, myr-GIIP, myr-GIIF, myr-GITI) is striking but not yet fully understood. Given the similarity of eluted peptides, which are of unknown origin, to the studied lipopeptides that may be linked to MDV infection, examining the functionality of YF1∗7.1 in the context of MDV mediated immunity will be the basis of future studies. While the contribution of YF1∗7.1 or more broadly of the MHC-Y cluster in eliciting an immune response is still unknown, some studies show evidence of changes in the MHC-Y genes expression levels during immune responses (45, 46). Also, MHC-Y genes variation is linked to MDV resistance (47) and resistance to Rous sarcoma-induced tumours (48).

One possibility is that myr-GII-like sequences are selectively captured by YF1∗7.1 proteins among all myristoylated proteins in cells. The crystal structure of the binary complexes offers some molecular basis supporting the specific capture hypothesis based in peptide specific binding interactions. This hypothesis can be addressed by future cellular capture studies with defined and total lipopeptides, along with consideration of the immunological nature of signals from proteins carrying this motif. This mechanism may be a critical part of how chickens detect and respond to viral infections, highlighting a unique aspect of avian immunity.

Collectively, our findings suggest that YF1∗7.1 and more broadly the MHC-I molecules encoded by the MHC-Y cluster of genes may play roles in the presentation of chicken viral lipopeptides that may be recognized by immune cognate receptors such as T cell receptors (TCRs) and Natural Killer (NK) cell receptors. Further, our study opens avenues for further research into the MHC-Y cluster of genes and the presentation of N-myristoylated lipopeptides derived from a variety of avian viruses. By understanding how these ligands function in various organisms, we may gain insights into the evolution of immune systems and develop better strategies for managing diseases in both animals and humans leading to improved vaccines and treatments for viral diseases, benefiting both agriculture and medicine.

## Materials and methods

### Cloning, expression, and purification of YF1∗7.1

The chicken YF1∗7.1 sequence harboring BirA and 6X-histidine affinity tags and the β2m genes were cloned into the pHLsec mammalian expression vector [19], and the protein was expressed for 7 days at 37 °C using HEK293S mammalian cells. The produced soluble YF1∗7.1 was then buffer exchanged against 10 mM Tris pH 8.0, 300 mM NaCl, and purified using Ni-NTA, size exclusion (Superdex S200 16/60 column) (GE Healthcare) and ion exchange (HitrapQ) chromatography techniques. Subsequently, the chicken YF1∗7.1 and β2m genes were also cloned into the bacterial pET30b expression vector, expressed in *E. coli* BL21 (DE3) cells and purified as inclusion bodies. As previously described, the inclusion bodies were solubilized in 10 mM Tris-HCl pH 8.0, 6 M guanidine hydrochloride, 1 mM DTT, 0.2 mM PMSF and 1 mM EDTA [20]. YF1∗7.1 (50 mg) and β2m (25 mg) were then *in vitro* refolded in 500 ml refolding buffer (0.1 M Tris-HCl pH 8.5, 0.4 M L-arginine, 0.5 mM oxidized glutathione, 5 mM reduced glutathione, and 5 M Urea), and in the presence of either myristoylated glycine (C14:0-gly; 2.5 mg) (Merck), or palmitoylated glycine (C16:0-gly; 2.5 mg) (Merck), or teg-1 (C14:0-GQAVS; 5 mg) (GL-Bio-chem), and or teg-2 (C14:0-GIIFS; 5 mg) (GL-Bio-chem). The refolding solution was then dialyzed against 10 mM Tris-HCl pH 8.0, and the refolded YF1∗7.1/β2m was purified by weak anion exchange (DEAE-Sepharose). The YF1∗7.1 was then buffer exchanged against 10 mM Tris pH 8.0, 150 mM NaCl, and further purified using size exclusion (Superdex S200 16/60 column) (GE Healthcare), and anion exchange chromatography (HiTrap Q column) methods.

### Crystallization and structure determination of YF1∗7.1-ligand binary complexes

Crystals of the mammalian expressed YF1∗7.1 (8–10 mg/ml) grew in 16% PEG 3350 and 200 mM NH_4_H_2_PO_4_ at 20 °C using the hanging drop vapor diffusion technique. The crystals were flash frozen in the mother liquor containing 8 to 15% glycerol as a cryoprotectant and data to 2.0 Å resolution were collected on the MX1 beamline at the Australian Synchrotron (Australia). Diffraction images were indexed and integrated using iMosflm, scaled, and merged using Aimless from the CCP4 suite (49, 50). YF1∗7.1 crystal structure was determined by molecular replacement using the program Phaser (51) and the previously solved YF∗7.1 structure as a search model (PDB code: 3P73) (11). An initial round of rigid body refinement was carried out with Refmac5 (52), followed by rounds of restrained refinement in Phenix refinement (53) and model building using Coot (54). Crystals of the YF1∗7.1 refolded *in vitro* with C14:0-gly grew in 25% PEG 3350, 0.2 M LiSO_4_, 0.1 M Bis-Tris pH 5.5, while the YF1∗7.1-C16:0-gly and YF1∗7.1-C14:0-teg-1 crystals were obtained in 20% PEG 4000, 0.2 M MgSO_4_, 10% Glycerol. YF1∗7.1-C14:0-teg-2 crystallized in 18% PEG 4000, Na acetate pH 4.5. These 4 datasets were collected on the MX2 beamline at the Australian Synchrotron (Australia) and processed using iMosflm and Aimless (49, 50), and their structured were determined as described above. The quality of the final structures was validated using the PDB validation server from the PDB database.

### Mass spectrometry analysis of YF1∗7.1 bound ligands

Both YF1∗7.1 and DR4 were analyzed side by side starting with the Bligh and Dyer lipid extraction method (13). Eluants in chloroform, methanol, and water were allowed to separate into organic and aqueous phases. The organic phase was collected, dried, redissolved, and stored in chloroform and methanol (1:1). For comparative lipidomics, both protein eluents were normalized to 20 μM based on the input protein concentration and injected three times for the normal phase HPLC-Quadrupole-Time-of-Flight (QTOF)-MS analysis according to a published method (55). Each sample was loaded onto an Inertsil Diol column (3 μm, 150 mm × 2.1 mm, GL Sciences) with a guard column (3 μm, 10 × 3.0 mm, GL Sciences) connected to the Agilent 1200 series HPLC system and Agilent 6520 Accurate-Mass Q-TOF. The CID-MS analysis was performed on either an Agilent 6520 or an Agilent 6546 Accurate-Mass Q-TOF instrument. The synthetic C18:1(N-Oleoyl) glycine standard was purchased from Millipore Sigma (#O9762).

## Data availability

The coordinates of the crystal structure of the YF1∗7.1-C14:0-gly (Mammalian-produced YF1∗7.1), YF1∗7.1-C14:0-gly (Bacterially-produced YF1∗7.1), YF1∗7.1-C16:0-gly (Bacterially-produced YF1∗7.1), YF1∗7.1-teg1 (C14:0) (Bacterially-produced YF1∗7.1) and YF1∗7.1-teg2 (C14:0) (Bacterially-produced YF1∗7.1) binary complexes were deposited in the Protein Data Bank (PDB) database under the accession code 9EB2, 9EB3, 9EB4, 9EB5 and 9EB6, respectively.

## Supporting information

This article contains supporting information.

## Conflict of interest

The authors declare that they have no conflicts of interest with the contents of this article.
