# Supplementary material for: Molecular basis for presentation of N-myristoylated peptides by the chicken YF1∗7.1 molecule[image]
Source: J Biol Chem. 2025 May 22;301(7):110253. doi: 10.1016/j.jbc.2025.110253 (PMC12212280; doi:10.1016/j.jbc.2025.110253)
Supplement: Supporting information [file mmc1.zip › Table-S1 2025_5_18.docx]

**Table S1. Data collection and refinement statistics**

|  | **YF1*7.1-C14:0-gly**  **(Mammalian)** | **YF1*7.1-C14:0-gly**  **(Bacterial)** | **YF1*7.1-C16:0-gly**  **(Bacterial)** | **YF1*7.1-teg1 (C14:0)** | **YF1*7.1-teg2 (C14:0)** |
| --- | --- | --- | --- | --- | --- |
| **Data collection** |  |  |  |  |  |
| Temperature | 100K | 100K | 100K | 100K | 100K |
| Resolution limits (Å) | 45.34-2.0 (2.07-2.0) | 37.9-2.3 (2.38-2.3) | 51.02-2.2 (2.28-2.2) | 39.39-2.08 (2.15-2.08) | 42.19-1.8 (1.84-1.8) |
| Space Group | P2_1_2_1_2_1_ | I121 | I121 | I121 | P12_1_1 |
| Cell dimensions (Å) | a=53.8, b=61.3, c=134.6  α=β=γ=90° | a=52.9, b=54.9, c=136.2  α=γ=90°, β=98.5° | a=56.6, b=55.4, c=133.4  α=γ=90°, β=100° | a=57.1, b=55.4, c=133.4  α=γ=90°, β=101.4° | a=55.3, b=55.6, c=65.3  α=γ=90°, β=97.9° |
| Total N^o.^ observations | 61668 (5986) | 34730 (3412) | 40472 (3959) | 49568 (4760) | 71908 (7104) |
| N^o.^ unique observations | 35868 (2208) | 17377 (1706) | 20758 (2036) | 24812 (2408) | 36579 (3645) |
| Multiplicity | 12 (10.5) | 6.8 (6.9) | 3.6 (3.5) | 6.3 (6) | 4 (4.1) |
| Data completeness | 99.7 (97.7) | 100 (100) | 100 (99) | 100 (97) | 99.9 (100) |
| Wilson B-factors (Å^2^) | 22.78 | 29.09 | 26.92 | 22.88 | 13.92 |
| CC_1/2_ | 0.999 | 0.994 | 0.994 | 0.998 | 0.999 |
| I/σ_I_ | 12.4 (3.6) | 8.1 (2.2) | 12.5 (2.5) | 11.0 (2.8) | 12.3 (3.4) |
| R_merge_ | (3.9) (11.2) | 6.0 (31.0) | 4.0 (9.8) | 4.6 (25.4) | 3.2 (31.9) |
| R_p.i.m_ ^1^ (%) | 4.5 (26.5) | 7.2 (34.3) | 5.5 (12.2) | 5.5 (28.7) | 4.3 (17.3) |
| **Refinement statistics** |  |  |  |  |  |
| R_factor_ ^2^ (%) | 16.2 | 18.0 | 15.7 | 18.6 | 17.1 |
| R_free_ ^3^ (%) | 20.9 | 23.6 | 22.0 | 24.3 | 22.1 |
| Non-hydrogen atoms   - Protein - Water - Heterogen | 371  239  20 | 371  117  30 | 368  166  27 | 371  201  23 | 371  444  15 |
| Ramachandran plot (%)   - Allowed region - Disallowed region | 100  0 | 100  0 | 100  0 | 100  0 | 100  0 |
| rmsd bonds (Å) | 0.002 | 0.003 | 0.013 | 0.003 | 0.004 |
| rmsd angles (°) | 0.6 | 0.68 | 1.18 | 0.67 | 0.69 |

^1^ R_p.i.m_ = Σ_hkl_ [1/(N-1)]^1/2^ Σi | I_hkl_, i - <I_hkl_> | / Σ_hkl_ <I_hkl_>

^2^ R_factor_ = ( Σ | |Fo| - |Fc| | ) / ( Σ |Fo| ) - for all data except as indicated in footnote 3.

**^3^** 5% of data was used for the R_free_ calculation.

Values in the parentheses refer to the highest resolution shell.
