# Supplementary material for: Molecular basis for presentation of N-myristoylated peptides by the chicken YF1∗7.1 molecule[image]
Source: J Biol Chem. 2025 May 22;301(7):110253. doi: 10.1016/j.jbc.2025.110253 (PMC12212280; doi:10.1016/j.jbc.2025.110253)
Supplement: Supporting information [file mmc1.zip › Table-S2.docx]

**Table S2. N-myristoyl-glycine (C14:0-gly) contacts with YF1*7.1 (Mammalian)**

| **YF1*7.1 residues** | **C14:0-gly atoms** | **Bond type** |
| --- | --- | --- |
|  | ***C14:0 myristoylated chain*** |  |
| Tyr7 | C3, C4 | VDW |
| Leu9 | C10 | VDW |
| Ile24 | C3 | VDW |
| Gly26 | C | VDW |
| Gly34 | C | VDW |
| Thr35 | C, C1 | VDW |
| Ala43 | C2 | VDW |
| Gln61 | C4, C5 | VDW |
| Lys64 | C5, C7, C9 | VDW |
| Ala65 | C5 | VDW |
| Gly68 | C9, C10 | VDW |
| Asp71 | C13, C14, C15 | VDW |
| Phe96 | C6, C8 | VDW |
| Tyr112^Oη^ | O | HB |
| Tyr112 | C13, O | VDW |
| Tyr149 | C15 | VDW |
| Trp153 | C11 | VDW |
|  | ***Glycine residue*** |  |
| Asp71^Oδ^ | N | HB |
| Asp71 | N | VDW |
| Asn75^Oδ2^ | O | HB |

HB: Hydrogen bond, VDW: Van der Waals. Cut-off at 4 Å for VDW interactions and 3.5 Å for HB.
