# Supplementary material for: Molecular basis for presentation of N-myristoylated peptides by the chicken YF1∗7.1 molecule[image]
Source: J Biol Chem. 2025 May 22;301(7):110253. doi: 10.1016/j.jbc.2025.110253 (PMC12212280; doi:10.1016/j.jbc.2025.110253)
Supplement: Supporting information [file mmc1.zip › Table-S4.docx]

**Table S4. N-palmitoyl-glycine (C16:0-gly) contacts with YF1*7.1**

| **YF1*7.1 residues** | **C16:0-gly atoms** | **Bond type** |
| --- | --- | --- |
|  | ***C16:0 myristoylated chain*** |  |
| Tyr7 | C4, C5 | VDW |
| Leu9 | C11 | VDW |
| Ile24 | C1 | VDW |
| Gly26 | C1 | VDW |
| Phe33 | C | VDW |
| Gly34 | C, C1 | VDW |
| Thr35 | C, C1, C2 | VDW |
| Tyr36 | C2 | VDW |
| Ala43 | C3 | VDW |
| Trp58 | C | VDW |
| Gln61 | C5, C6 | VDW |
| Lys64 | C6, C9 | VDW |
| Ala65 | C6 | VDW |
| Gly68 | C11 | VDW |
| Asp71 | C14, C15, C16 | VDW |
| Met94 | C13 | VDW |
| Phe96 | C7, C10 | VDW |
| Tyr112^Oη^ | O | HB |
| Tyr112 | C15, O | VDW |
| Tyr149 | O | VDW |
| Arg152 | C17, O1 | VDW |
| Trp153 | C10, C12, C17, O1, O2 | VDW |
|  | ***Glycine residue*** |  |
| Asp71^Oδ^ | N | HB |
| Asp71 | N, O2 | VDW |
| Tyr149 | O1 | VDW |
| Arg152^Nη1^ | O1, O2 | HB |
| Arg152^Nη2^ | O1 | HB |
| Arg152 | O1 | VDW |
| Trp153^Nε1^ | O1 | HB |

HB: Hydrogen bond, VDW: Van der Waals. Cut-off at 4 Å for VDW interactions and 3.5 Å for HB.
