# Supplementary material for: Molecular basis for presentation of N-myristoylated peptides by the chicken YF1∗7.1 molecule[image]
Source: J Biol Chem. 2025 May 22;301(7):110253. doi: 10.1016/j.jbc.2025.110253 (PMC12212280; doi:10.1016/j.jbc.2025.110253)
Supplement: Supporting information [file mmc1.zip › Table-S5.docx]

**Table S5. N-myristoylated teg-1(C14:0) contacts with YF1*7.1**

| **YF1*7.1 residues** | **C14:0-teg-1 atoms/residues** | **Bond type** |
| --- | --- | --- |
|  | ***C14:0 myristoylated chain*** |  |
| Tyr7 | C10, C11 | VDW |
| Leu9 | C5 | VDW |
| Gly26 | C14 | VDW |
| Gly34 | C14 | VDW |
| Thr35 | C13, C14 | VDW |
| Ala43 | C13 | VDW |
| Gln61 | C9, C10 | VDW |
| Lys64 | C6, C7, C8, C9 | VDW |
| Ala65 | C7, C9 | VDW |
| Gly68 | C4, C5 | VDW |
| Asp71 | C1, C2 | VDW |
| Met94 | C3, O | VDW |
| Tyr112^Oη^ | O | HB |
| Tyr112 | O | VDW |
| Trp153 | C3, C4, C5 | VDW |
|  | ***Peptide*** |  |
| Asp71^Oδ1^ | Gly1^N^ | HB |
| Asp71 | Gly1, Gln2 | VDW |
| Trp74^Nε1^ | Gln2^Oε1^ | HB |
| Trp74 | Gln2, Ala3 | VDW |
| Asn75^Nδ2^ | Gly1^O^ | HB |
| Asn75^Oδ1^ | Ala3^N^ | HB |
| Asn75 | Gly1, Gln2, Ala3 | VDW |
| Arg82^Nη1^ | Ala3^O^ | HB |
| Arg82^Nη2^ | Ala3^O^, Val4^O^ | HB |
| Arg82 | Ala3, Val4, Ser5 | VDW |
| Phe119 | Ala3 | VDW |
| Ile138 | Ser5 | VDW |
| Thr139^Oγ1^ | Val4^O^ | HB |
| Thr139 | Ala3, Val4 | VDW |
| Arg142^Nε^ | Ser5^O^ | HB |
| Arg142 | Val4, Ser5 | VDW |
| Trp143^Nε1^ | Gln2^O^ | HB |
| Trp143 | Gln2, Ala3, Val4 | VDW |
|  |  |  |

HB: Hydrogen bond, VDW: Van der Waals. Cut-off at 4 Å for VDW interactions and 3.5 Å for HB.
