# Supplementary material for: Molecular basis for presentation of N-myristoylated peptides by the chicken YF1∗7.1 molecule[image]
Source: J Biol Chem. 2025 May 22;301(7):110253. doi: 10.1016/j.jbc.2025.110253 (PMC12212280; doi:10.1016/j.jbc.2025.110253)
Supplement: Supporting information [file mmc1.zip › Table-S6.docx]

**Table S6. N-myristoylated teg-2 (C14:0) contacts with YF1*7.1**

| **YF1*7.1 residues** | **C14:0-teg-2 atoms/residues** | **Bond type** |
| --- | --- | --- |
|  | ***C14:0-myristoylated chain*** |  |
| Tyr7 | C11 | VDW |
| Leu9 | C4, C5 | VDW |
| Ile24 | C7, C8 | VDW |
| Gly34 | C14 | VDW |
| Thr35 | C13, C14 | VDW |
| Ala43 | C12 | VDW |
| Gln61 | C10 | VDW |
| Lys64 | C6, C7, C9 | VDW |
| Ala65 | C7 | VDW |
| Gly68 | C4 | VDW |
| Asp71 | C1, C2 | VDW |
| Met94 | C3, O | VDW |
| Phe96 | C5 | VDW |
| Tyr112^Oη^ | O | HB |
| Tyr112 | O | VDW |
|  | ***Peptide*** |  |
| Asp71^Oδ2^ | Gly1^N^ | HB |
| Asp71 | Gly1, Ile2 | VDW |
| Trp74^Nε1^ | Phe4^O^ | HB |
| Trp74 | Ile2, Phe4, Ser5 | VDW |
| Asn75^Oδ1^ | Ile3^N^, Phe4^N^ | HB |
| Asn75^Nδ2^ | Gly1^O^ | HB |
| Asn75 | Gly1, Ile2, Ile3, Phe4 | VDW |
| Arg78 | Phe4, Ser5 | VDW |
| Leu79 | Phe4 | VDW |
| Pro80 | Phe4 | VDW |
| Met92 | Ile3 | VDW |
| Tyr112 | Ile3 | VDW |
| Phe119 | Ile3, Phe4 | VDW |
| Thr139^Oγ1^ | Ile3^O^ | HB |
| Thr139 | Ile3 | VDW |
| Trp143^Nε1^ | Ile2^O^ | HB |
| Trp143 | Ile2, Ile3 | VDW |
| Trp153 | C3 | VDW |

HB: Hydrogen bond, VDW: Van der Waals. Cut-off at 4 Å for VDW interactions and 3.5 Å for HB.
