# Supplementary material for: Molecular basis for presentation of N-myristoylated peptides by the chicken YF1∗7.1 molecule[image]
Source: J Biol Chem. 2025 May 22;301(7):110253. doi: 10.1016/j.jbc.2025.110253 (PMC12212280; doi:10.1016/j.jbc.2025.110253)
Supplement: Supporting information [file mmc1.zip › Supplementary_material_text.docx]

**Supplementary materials**

**Molecular basis for presentation of N-myristoylated peptides**

**by the chicken YF1*7.1 molecule**

Yogesh Khandokar^1,φ,*^, Tan Yun Cheng^2,*^, Carl J. H. Wang^1,†^, Thinh-Phat Cao^1^, Raghavendra S. K. Nagampalli^1,‡^, Komagal Kannan Sivaraman^1^, Ildiko Van Rhijn^2,3,4^, Jamie Rossjohn^1,5,#^, D. Branch Moody^2#^, and Jérôme Le Nours^1,#^

^1^Infection and Immunity Program and Department of Biochemistry and Molecular Biology, Biomedicine Discovery Institute, Monash University, Clayton, Victoria 3168, Australia.

^2^ Division of Rheumatology, Inflammation and Immunity, Brigham and Women’s Hospital, Harvard Medical School, Boston, MA, USA.

^3^Department of Infectious Diseases and Immunology, Faculty of Veterinary Medicine, Utrecht University, Utrecht, The Netherlands.

^4^Department of Medical Biology, Amsterdam University Medical Center, Amsterdam, The Netherlands.

^5^Institute of Infection and Immunity, Cardiff University, School of Medicine, Heath Park, Cardiff CF14 4XN, UK.

^φ^ Present address: Australian Synchrotron-ANSTO, Clayton, Victoria 3168, Australia.

^†^Present address: Institute for Protein Design, University of Washington, Seattle, WA, USA.

^‡^ Present address: St Jude Children's Research Hospital, Department of Immunology, Memphis, TN, USA.

* joint 1^st^ authors

# joint senior and corresponding authors : [Jamie.rossjohn@monash.edu](mailto:Jamie.rossjohn@monash.edu), [bmoody@bwh.harvard.edu](mailto:bmoody@bwh.harvard.edu), [jerome.lenours@monash.edu](mailto:jerome.lenours@monash.edu)

**Supplementary Figures.**

**Fig. S1. (A)** Superposition of the mammalian expressed YF1*7.1 is shown in white with the refolded YF1*7.1 protein (bacterially produced) is shown in light green (Pdb code: 3P77) (11). The bound N-myristoyl-glycine (C14:0) and the PEG molecules are shown in red and light green sticks, respectively. **(B)** Fo-Fc electron density unbiased omit map contoured at 2.2σ level of N-myristoyl-glycine (C14:0) in the binding pocket of YF1*7.1 is shown. **(C)** Fo-Fc electron density unbiased omit map contoured at 2.2σ level of N-myristoyl-glycine (C14:0) bound in the refolded YF1*7.1 protein (bacterially produced). **(D)** Fo-Fc electron density map contoured at 2.2σ level of N-myristoyl-glycine (C16:0) bound in the refolded YF1*7.1. **(E)** Molecular interactions of N-myristoyl-glycine (C14:0) with the refolded YF1*7.1 protein (bacterially produced) are shown. **(F)** Molecular interactions of N-myristoyl-glycine (C16:0) with refolded YF1*7.1 are shown.

**Fig. S2. (A)** Fo-Fc electron density (unbiased omit map) contoured at 2.2σ level of C14:0-teg-1 bound in the refolded YF1*7.1 protein (bacterially produced). **(B)** Fo-Fc electron density (unbiased omit map) contoured at 2.2σ level of C14-teg-2 bound in the refolded YF1*7.1 protein (bacterially produced). **(C)** Molecular interactions of YF1*7.1 with the glycine moiety of C14:0-teg-1. **(D)** Molecular interactions of YF1*7.1 with the glycine moiety of C14:0-teg-2. Hydrogen bonds are shown as yellow dashed lines in **(C)** and **(D)**.

**Fig. S3.** Analysis of the buried and exposed surface area (Å^2^) of the C14 and amino acid side chain residues of Teg-1 **(A)** and Teg-2 **(B)** N-myristoylated peptides. The value (Å^2^) of the buried and exposed surface areas are depicted as black and white columns, respectively.
