# Supplementary figures and images for: Molecular basis for presentation of N-myristoylated peptides by the chicken YF1∗7.1 molecule[image]
Source: J Biol Chem. 2025 May 22;301(7):110253. doi: 10.1016/j.jbc.2025.110253 (PMC12212280; doi:10.1016/j.jbc.2025.110253)

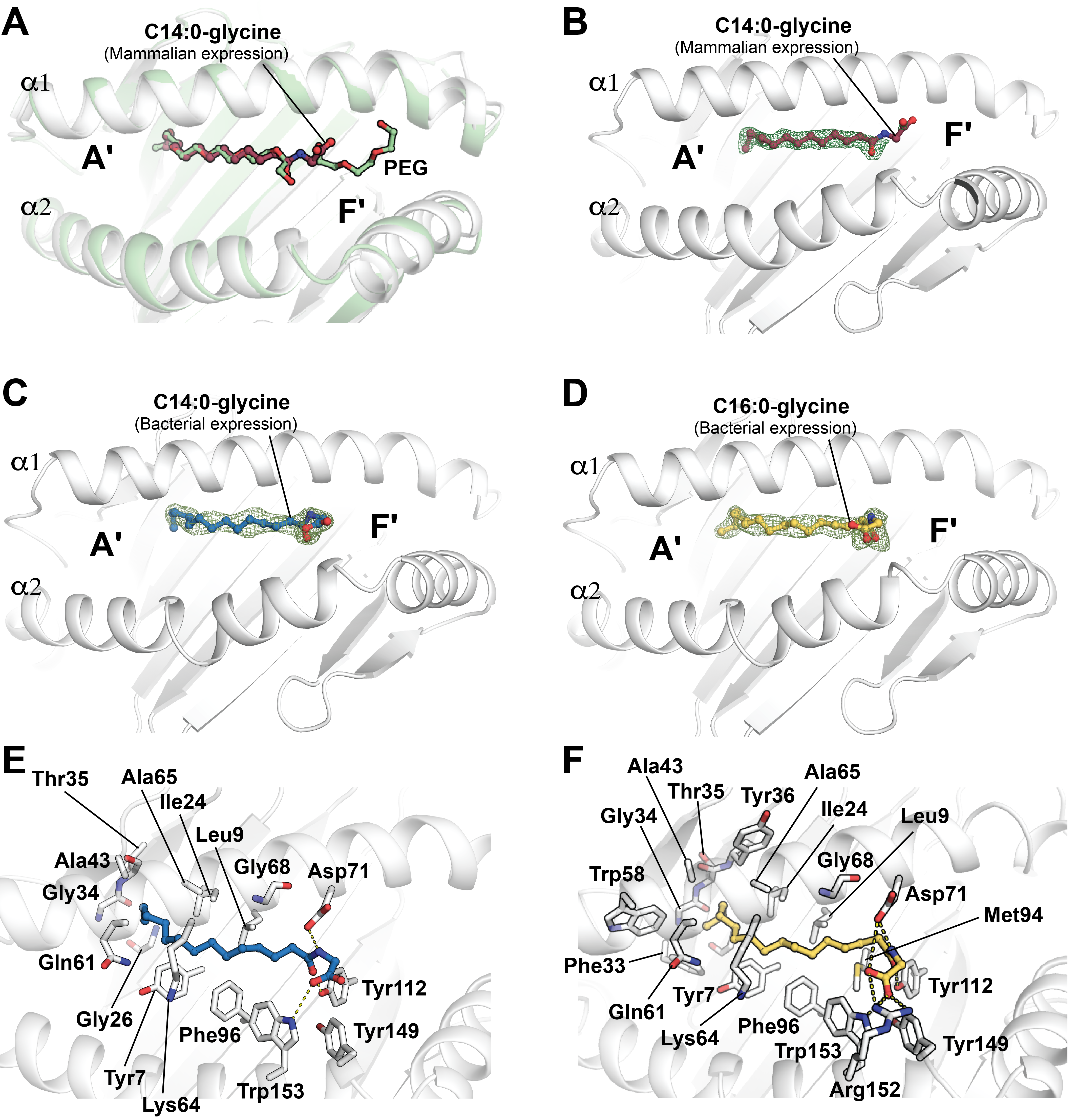

Supplement: Supporting information [file mmc1.zip › Figure.S1.tif]

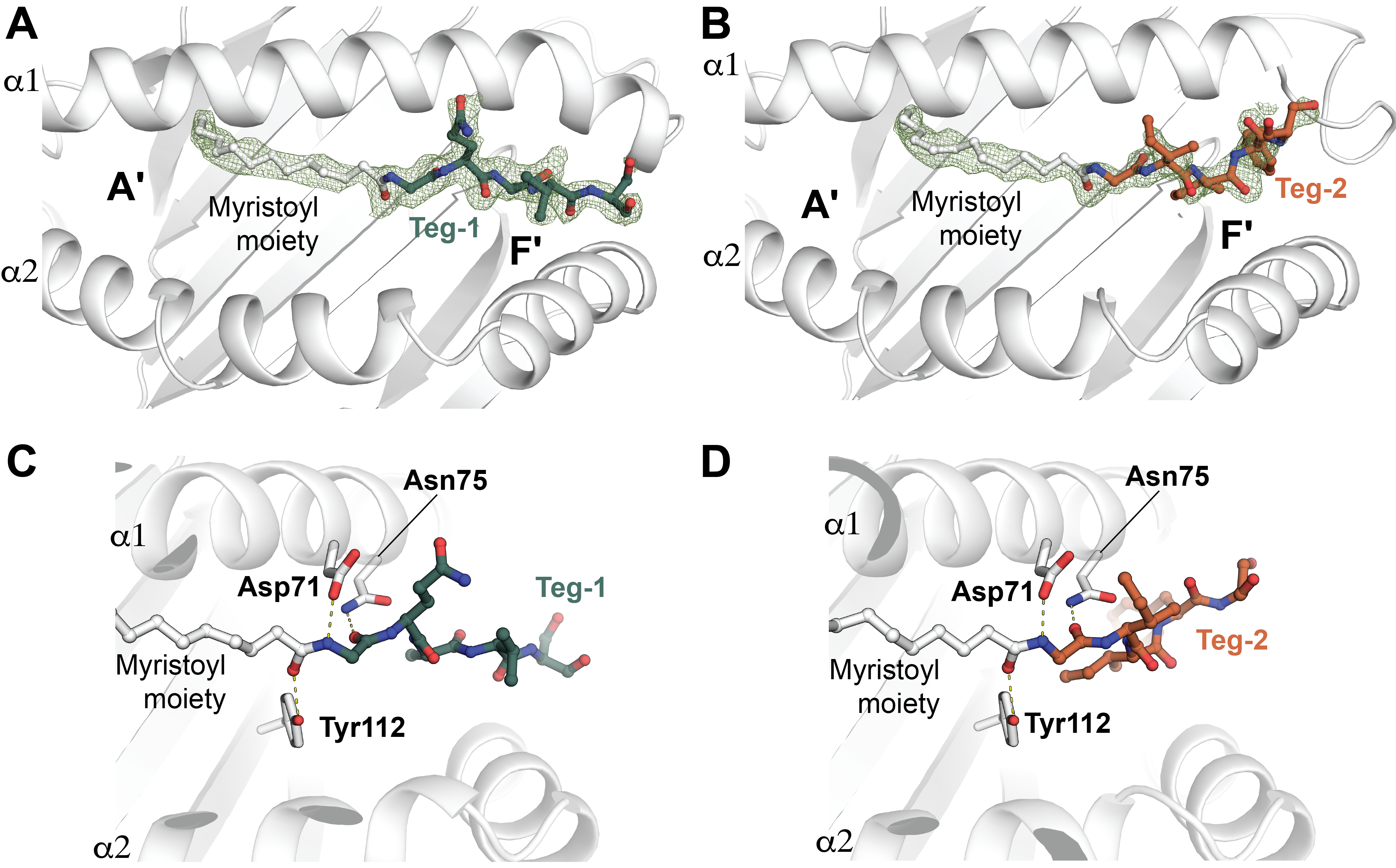

Supplement: Supporting information [file mmc1.zip › Figure.S2.tif]

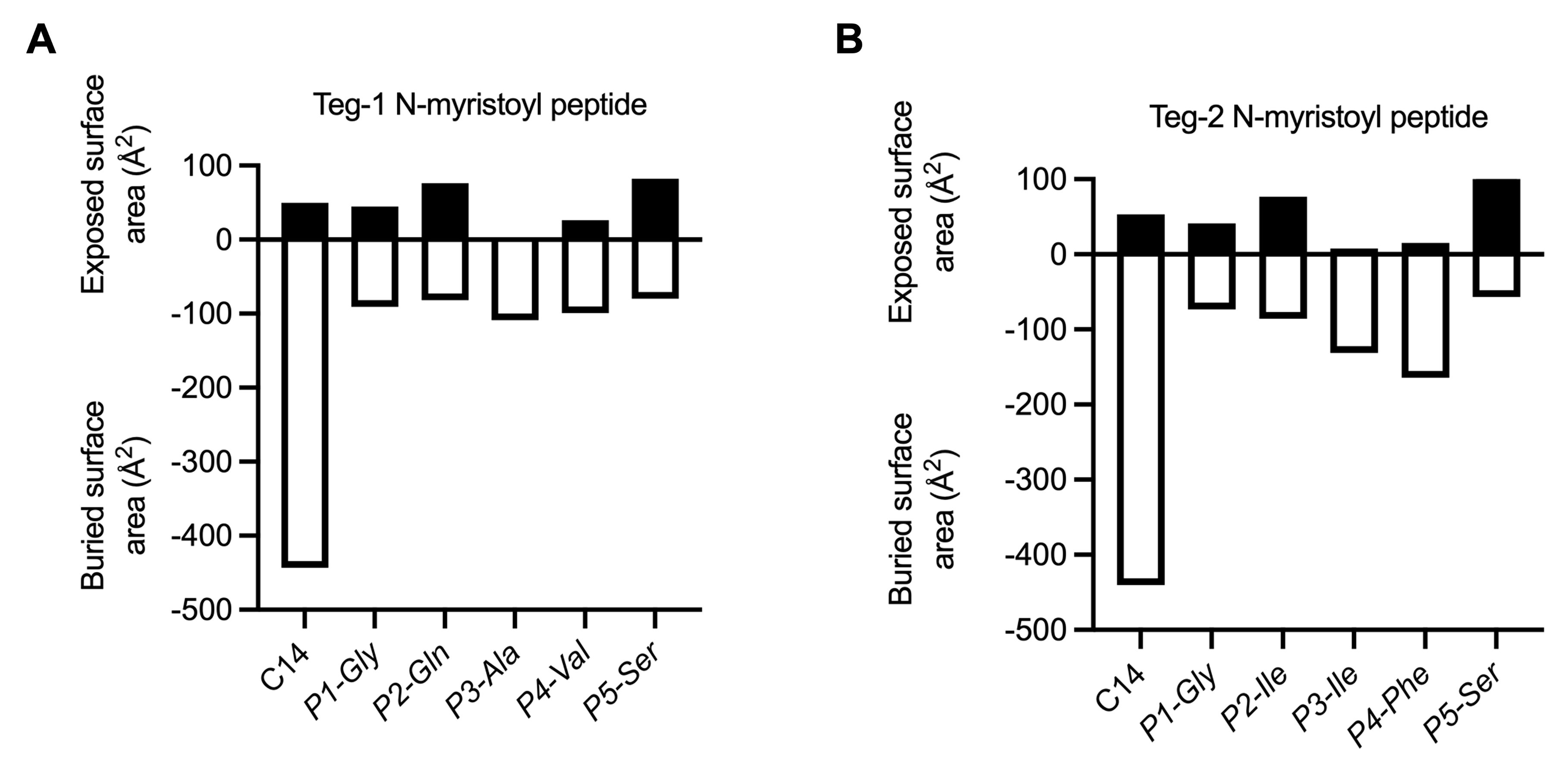

Supplement: Supporting information [file mmc1.zip › Figure.S3.tif]
